# Abnormalities of regional brain function in Parkinson’s disease: a meta-analysis of resting state functional magnetic resonance imaging studies

**DOI:** 10.1038/srep40469

**Published:** 2017-01-12

**Authors:** PingLei Pan, Yang Zhang, Yi Liu, He Zhang, DeNing Guan, Yun Xu

**Affiliations:** 1 Department of Neurology, Drum Tower Hospital, Medical School of Nanjing University, Nanjing, PR China; 2 Department of Neurology, The Affiliated Yancheng Hospital, School of Medicine, Southeast University, Yancheng, PR China; 3 The State Key Laboratory of Pharmaceutical Biotechnology, Nanjing University, Nanjing, Jiangsu, PR China; 4 Jiangsu Key Laboratory for Molecular Medicine, Nanjing University Medical School, Nanjing, Jiangsu, PR China; 5 Jiangsu Province Stroke Center for Diagnosis and Therapy, Nanjing, PR China; 6 Nanjing Neuropsychiatry Clinic Medical Center, Nanjing, PR China

## Abstract

There is convincing evidence that abnormalities of regional brain function exist in Parkinson’s disease (PD). However, many resting-state functional magnetic resonance imaging (rs-fMRI) studies using amplitude of low-frequency fluctuations (ALFF) have reported inconsistent results about regional spontaneous neuronal activity in PD. Therefore, we conducted a comprehensive meta-analysis using the Seed-based *d* Mapping and several complementary analyses. We searched PubMed, Embase, and Web of Science databases for eligible whole-brain rs-fMRI studies that measured ALFF differences between patients with PD and healthy controls published from January 1st, 2000 until June 24, 2016. Eleven studies reporting 14 comparisons, comparing 421 patients and 381 healthy controls, were included. The most consistent and replicable findings in patients with PD compared with healthy controls were identified, including the decreased ALFFs in the bilateral supplementary motor areas, left putamen, left premotor cortex, and left inferior parietal gyrus, and increased ALFFs in the right inferior parietal gyrus. The altered ALFFs in these brain regions are related to motor deficits and compensation in PD, which contribute to understanding its neurobiological underpinnings and could serve as specific regions of interest for further studies.

Parkinson’s disease (PD) is a common neurodegenerative disorder associated with progressive disability and chronic suffering that lead to a great social burden^1^,^2^. PD is traditionally defined as a movement disorder resulting from a prominent loss of dopaminergic neurons of the nigrostriatal pathway, but more recently it has been demonstrated that widespread non-motor symptoms, such as cognitive impairment and mood disorders, are also prevalent, which involve extensive brain regions^3^,^4^,^5^,^6^. PD is clinically and etiologically heterogeneous and its complex neurobiological underpinnings remain to be fully elucidated^3^.

During the last decade, resting-state functional magnetic resonance imaging (rs-fMRI) has become an established approach for exploring functional neuroanatomy *in vivo* and numerous studies have sought to unravel the key abnormalities of brain function involved in the pathophysiology of PD^7^. Amplitude of low-frequency fluctuations (ALFF), an index to measure changes in resting-state blood oxygen level dependent (BOLD) signals, has been shown to reflect regional spontaneous neuronal activity^8^. ALFF has been widely used to explore regional changes of brain function in neuropsychiatric disorders^9^,^10^,^11^,^12^,^13^,^14^. Aberrant ALFF patterns in PD have been shown to be related to motor subtypes^15^, motor severity^16^,^17^, disease progression^17^,^18^, apathy^16^, depression^16^,^19^,^20^,^21^, and visual hallucinations^22^. These studies indicate that PD pathophysiology is involved in widespread abnormalities of regional spontaneous neuronal activity beyond those within the motor network. Although ALFF studies have substantially enhanced our understanding of the neural substrates underlying PD, conclusions from these studies have not been entirely consistent, raising questions about their replicability and reliability. Widespread and heterogeneous ALFF abnormalities in many brain regions, such as the motor cortices, striatum, cerebellum, and brain stem, as well as frontal, temporal, parietal, occipital, and cingulate cortices (see Supplementary Table 1)^13^,^15^,^17^,^18^,^19^,^22^,^23^,^24^,^25^,^26^,^27^, were observed across studies in patients with PD in comparison with healthy controls. Differences in sample size, disease severity, disease duration, medication status, and imaging methodology may partially contribute to these inconsistencies. For example, ALFF differences about effect of therapy were observed in patients with PD^23^,^25^. Thus, to overcome the inconsistences across single ALFF studies is very timely and necessary.

We aimed at conducting a quantitative and voxel-based meta-analysis of ALFF changes in patients with PD. In addition, we set out to perform meta-regression analyses to examine the confounding effects of demographics and clinical variables on ALFF changes in PD. Furthermore, several complementary analyses of jackknife sensitivity, heterogeneity, and publication bias were performed to explore the most consistent and reliable findings. Here, we used Seed-based *d* Mapping (SDM), a well validated meta-analytic tool for coordinate-based neuroimaging data^28^,^29^,^30^,^31^,^32^,^33^. SDM has already been applied to identify reliable brain anatomical or functional alterations in many neuropsychiatric disorders including Alzheimer’s disease^34^,^35^, PD^36^, multiple sclerosis^37^,^38^, amyotrophic lateral sclerosis^29^,^39^, depression^30^,^40^, and others^28^,^33^.

## Results

### Included studies and sample characteristics

**Figure 1:**
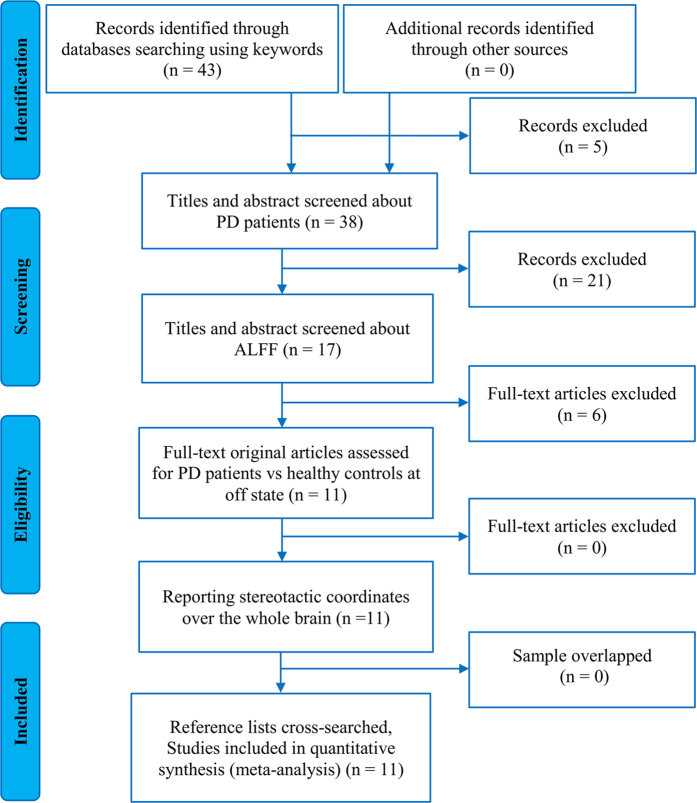
Flow diagram for inclusion/exclusion of studies. **Key:** PD, Parkinson’s disease; ALFF, amplitude of low-frequency fluctuations.

Figure 1 showed the flow diagram for inclusion/exclusion of studies in the meta-analysis. The systematic search yielded a total of 43 relevant documents. After initially screen of the titles and abstracts, 17 ALFF studies were potentially eligible for this meta-analysis. Of these, 6 studies were excluded because of the following reasons: one was an abstract^41^; one used a method of regions of interest^42^; one applied an approach of support vector machine training^43^; one did not perform a direct comparison between PD patients and healthy controls^16^; and two just reported findings from the on-state of PD patients^22^,^44^. The remaining 11 studies were included in the meta-analysis. Of these, two studies reported both on- and off-state results, only the latter datasets were included^23^,^25^. One study reported the baseline and follow-up findings, only the former dataset was included^17^. Two studies reported two datasets, respectively with non-depressed and depressed PD, and only the non-depressed datasets were included^19^,^21^. Another two studies reported two distinct^15^ and three distinct datasets^18^, respectively, and all of them were included. Totally, 11 original studies reporting 14 datasets were finally included in this meta-analysis^13^,^15^,^17^,^18^,^19^,^21^,^23^,^24^,^25^,^26^,^27^. These included datasets reported ALFF differences between 421 patients with PD (232 males and 189 females; mean age = 59.43 years) and 381 healthy controls (216 males and 165 females; mean age = 59.77 years). There were no significant differences between patients with PD and healthy controls regarding age (standardized mean difference = 0.018; 95% confidence interval = −0.114 to 0.151, z = 0.27, p = 0.786) or gender distribution (relative risk = 0.995, 95% CI = 0.925 to 1.070, z = 0.14, p = 0.891). Quality assessment indicated that the quality of these studies was acceptable because its score of each study was no less than 18 (total score = 20) (Table 1). The demographic, clinical, and imaging characteristics of each study included in this meta-analysis are summarized in Table 1.

### ALFF differences of the voxel-wise meta-analysis

**Figure 2:**
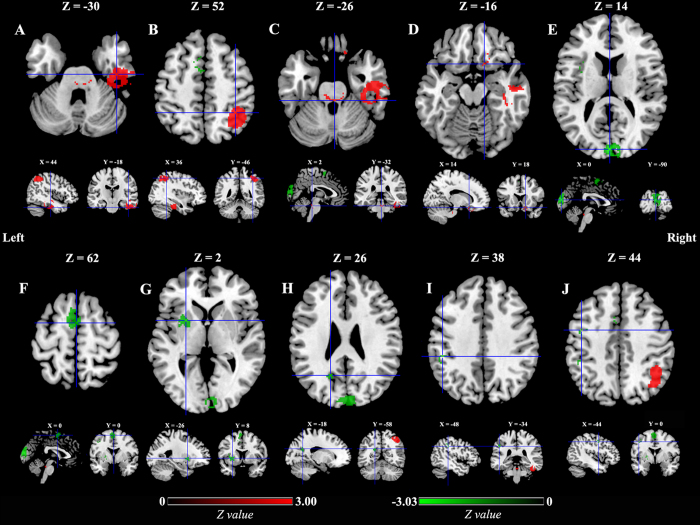
ALFF differences in patients with PD compared to healthy controls. **Key:** Red and green colors indicate increased and decreased ALFFs in patients with PD compared to healthy controls, respectively. ALFF, amplitude of low-frequency fluctuations; (**A**), Right inferior temporal/middle temporal/fusiform/parahippocampal gyri; (**B**), Right inferior parietal gyrus; (**C**), Brainstem (pons and midbrain); (**D**), Right orbitofrontal cortex; (**E**), Left/Right cuneus cortices; (**F**), Left/Right supplementary motor areas; (**G**), Left putamen; (**H**), Left cuneus cortex; (**I**), Left inferior parietal gyrus; (**J**), Left lateral premotor cortex.

As shown in Figure 2, the voxel-wise meta-analysis identified increased ALFFs in the right inferior temporal gyrus extending to the middle temporal, fusiform, and parahippocampal gyri, right inferior parietal gyrus, right inferior parietal gyrus, brainstem (pons extending to midbrain), and right orbitofrontal cortex in patients with PD compared to healthy controls. In contrast, decreased ALFFs were observed in the bilateral cuneus cortices, bilateral cuneus cortices, bilateral supplementary motor areas (SMAs), left putamen, left inferior parietal gyrus, and left lateral premotor cortex. The details of the results are presented in Table 2.

### Analyses of jackknife sensitivity, heterogeneity, and publication bias

The jackknife sensitivity analysis (Table 3) revealed that regions of ALFF differences in the right inferior temporal gyrus extending to the middle temporal, fusiform, and parahippocampal gyri, right inferior parietal gyrus, and bilateral cuneus cortices (BAs 18, 17, and 19) were replicable in all 14 datasets. Regions of ALFF differences in the brainstem, right orbitofrontal cortex, bilateral SMAs, left putamen, left inferior parietal gyrus, and left lateral premotor cortex were replicable in at least 12 datasets. While a brain region in the left cuneus cortex (BA 23) was only replicable in 10 datasets. The heterogeneity analysis revealed significant unexplained between-study variability of ALFF changes in widespread brain regions, including the left superior temporal gyrus extending to the middle temporal gyrus and insula, right caudate nucleus, right fusiform gyrus extending to the inferior temporal gyrus, right cerebellum (lobule VI), brainstem (pons), left cerebellum (crus I), right striatum, right inferior frontal gyrus, left superior occipital gyrus, left cuneus cortex (BA 23), right inferior temporal gyrus, right cerebellum (lobule IV/V), and left posterior cingulate cortex (PCC) (Supplementary Table 3). In addition, publication bias examined by the Egger’s test was detected in the regions of ALFF differences in the right inferior temporal gyrus extending to middle temporal, fusiform, and parahippocampal gyri (p < 0.001), brainstem (p = 0.046), right orbitofrontal cortex (p = 0.018), and bilateral cuneus cortices (p = 0.002). No publication bias was identified in other regions obtained from the voxel-wise meta-analysis (Table 2).

### Meta-regression analyses

Meta-regression analysis revealed that the PD group with older mean age (available in all datasets) exhibited greater ALFFs in the right inferior temporal gyrus extending to the middle temporal, fusiform, and parahippocampal gyri, right inferior parietal gyrus and lesser ALFFs in the bilateral cuneus cortices. Higher average H&Y stage in the PD group (available in 10 datasets) was associated with greater ALFFs in the bilateral precuneus/PCC and right inferior parietal gyrus. Higher average UPDRS-III score in the PD group (available in all datasets) correlated with greater ALFFs in the right inferior parietal gyrus and bilateral precuneus/PCC, and lesser ALFFs in the bilateral SMAs. Regression analysis indicated that longer mean illness duration of PD patients (available in 13 datasets) was associated with greater ALFFs in the right fusiform/inferior temporal gyri. The results of these meta-regression analyses are listed in Table 4.

## Discussion

In the present study, we conducted a meta-analysis to identify the most consistent and reliable ALFF changes in PD. Besides the main voxel-based meta-analysis, several complementary analyses, such as jackknife sensitivity, heterogeneity, and publication bias analyses were performed to explore the robustness of the findings. These comprehensive analyses showed that decreased ALFFs in the bilateral SMAs, left putamen, left lateral premotor cortex, and left inferior parietal gyrus and increased ALFFs in the right inferior parietal gyrus were the most consistent and reliable findings in patients with PD compared to healthy controls. Further meta-regression analyses indicated an effect of motor severity on ALFF changes in the bilateral SMAs and right inferior parietal gyrus, and illness severity on ALFF changes in the right inferior parietal gyrus.

Cardinal motor impairments characterize PD and dysfunction of the cortico-striatal-thalamic-cortical motor circuits is a fundamental model implicated in its pathophysiology^45^. Our meta-analysis consistently identified decreased ALFFs in the left putamen, bilateral SMAs, and left lateral premotor cortex. Decreased ALFFs are considered to reflect local spontaneous neuronal hypoactivity in these regions and indicate disease-related functional deficits secondary to degeneration of the dopaminergic neurons of the nigrostriatal system^45^,^46^. Neuronal neurodegeneration, dopaminergic deficits, hypo-metabolism, and hypoactivation of the putamen have been convergently confirmed in PD^45^,^47^,^48^,^49^,^50^,^51^,^52^,^53^,^54^. The putamen has dense anatomical and functional connections with motor cortical areas in normal humans^55^,^56^,^57^. While less functional connectivity between the putamen and cortical motor areas was observed in PD patients^58^,^59^.

Hypoactivity in the SMA in our meta-analysis is well in line with previous task-related and resting-state fMRI, and positron emission tomography (PET) studies^51^,^52^,^60^,^61^,^62^ and could be modulated by dopaminergic treatment in patients with PD^52^,^61^,^63^. However, brain activation (hypoactivity or hyperactivity) in the lateral premotor cortex has not been always consistent in task-related fMRI or PET studies^62^,^63^,^64^,^65^,^66^. A recent meta-analysis including 24 fMRI and PET studies in PD patients with motor tasks even could not detect consistent cortical activation in the lateral premotor cortex^67^. The activation inconsistences may attribute to task-specific recruitment of cortical motor areas or heterogeneity in task performance performed in enrolled patients. Rs-fMRI avoids any confound of variable task performance and can be easily implemented to obtain relatively stable results. The SMA is well known to play a critical role in internal motor preparation and execution^63^,^68^, whereas the lateral premotor cortex is involved more with externally guided movements^69^. SMA is a therapeutic target for PD management and repetitive transcranial magnetic stimulation over the SMA has been shown to be effective for improving motor symptoms^70^,^71^. Moreover, our meta-analysis revealed that severer motor deficits in the PD group correlated with lesser ALFFs in the bilateral SMAs. Whether ALFF changes in the SMAs could serve as a prognostic imaging marker merits further investigations. Our meta-analysis together with previous studies suggests that resting-state hypoactivation in the putamen, SMA, and premotor may contribute to the cardinal motor signs underlying PD.

Interestingly, the present study observed an asymmetrical activation in the inferior parietal cortex. Analysis of anatomical connectivity using probabilistic tractography shows that the rostral inferior parietal areas were predominantly connected to inferior frontal, motor, premotor, and somatosensory areas involved in higher motor functions, whereas the caudal areas are more strongly connected with posterior parietal, higher visual and temporal areas related to spatial attention and language processing^72^,^73^,^74^. Our meta-analysis identified hypoactivation in the left rostral inferior parietal cortex (BA 40) and hyperactivation in the right caudal inferior parietal cortex (mainly BA39) with a differential functional involvement. Previous task-related fMRI or PET studies also observed abnormal activation or connectivity of the rostral inferior parietal cortex (BA 40) in patients with PD^65^,^66^,^69^,^75^. In addition, rs-fMRI data in PD patients showed abnormal functional connectivity between the putamen and the inferior parietal cortex (BA 40)^76^. Whereas the caudal inferior parietal cortex (BA39) is an important hub of the default mode network (DMN), which is one of the most investigated resting-state networks, involved in higher cognitive processes^77^. Although heterogenous in the meta-analysis, other regions such as the right inferior temporal gyrus extending to the middle temporal, fusiform, and parahippocampal gyri, and precuneus/PCC observed were also the components of the DMN^77^,^78^. Tessitore *et al*. found a decreased resting-state functional connectivity of the right medial temporal lobe and bilateral inferior parietal cortex within the DMN even in cognitively unimpaired patients with PD^79^. Furthermore, they showed significant positive correlations between this decreased DMN connectivity and cognitive parameters such as memory test and visuospatial scores^79^. Regional spontaneous hyperactivation in the caudal inferior parietal cortex may reflect a compensatory way for the remote decreased functional connectivity. However, regional hyperactivation in this region may compensate more for the motor impairment in PD patients because our meta-analyses indicated that both increased motor severity and illness severity correlated with greater hyperactivation in this area.

Other regions are highly heterogenous or not robust detected in the meta-analysis. Besides the unexplained between-study variability of ALFF changes in the right inferior temporal gyrus extending to the middle temporal, fusiform, and parahippocampal gyri (BA 20), brainstem (pons and midbrain), and left cuneus cortex (BA 23), several moderator variables contributed to understanding the source of the heterogeneity. Of these, mean age in the PD group had a significant effect on the ALFF changes in the right inferior temporal gyrus extending to the middle temporal, fusiform, and parahippocampal gyri (BA 20), and bilateral cuneus cortices (BAs 18 and 17). In addition, we noted that the PD group with longer mean illness duration tended to have greater ALFF changes in the right fusiform extending to the inferior temporal gyri (BA 20). We also observed that decreased ALFFs in the left cuneus cortex (BA 23) were replicable only in 10 out of the 14 datasets as revealed by the jackknife sensitivity analysis. Unlike other coordinate-based meta-analytic methods for neuroimaging data, such as the activationlikelihoodestimation^67^,^80^ and multilevel kernel density analyses^81^,^82^, the SDM offers comprehensive information about robustness of the findings^28^,^30^,^83^. The present study suggests that future meta-analysis of neuroimaging data would benefit from complementary analyses to minimize the risk of false positives^30^,^83^.

### Limitations and perspectives

Despite the strengths, several limitations of the present meta-analysis should be acknowledged. First, SDM is a coordinate-based rather than an image-based meta-analytic method, which might lead to less accurate results that is also inherent to other coordinate-based meta-analytic approaches^28^,^80^,^82^,^83^. However, it is more difficult to obtain original imaging data than reported peak coordinates from original studies. Second, some potential methodological heterogeneity, such as different image analytical procedures (frequency differences, ALFF and fractional ALFF analyses)^17^,^23^,^24^,^26^ existed across studies. We could not further conduct subgroup analyses because of the limited number of eligible studies for controlling these moderator variables. Third, the ALFF studies in our meta-analyses mainly included Chinese samples, which might limit the findings to other populations. Further studies with more efforts are warranted to apply the ALFF method to other samples. Fourth, although ALFF is thought to reflect regional spontaneous neuronal activity, its exact neurobiological basis remains to be fully elucidated^84^. Future studies with multimodal imaging techniques and analysis approaches may provide more insights about the function-structure associations of the PD brain^85^.

## Conclusions

Using the comprehensive meta-analytic approach, we identified the most consistent and reliable ALFF changes, including decreased ALFFs in the bilateral SMAs, left putamen, left premotor cortex, and left inferior parietal gyrus, and increased ALFFs in the right inferior parietal gyrus in patients with PD compared to healthy controls. The altered ALFFs in these brain regions may be related to motor deficits and compensation in PD, which could serve as specific regions of interest for further studies. Other highly heterogenous or not robust regions, such as right inferior temporal gyrus extending to the middle temporal, fusiform and parahippocampal gyri, brainstem (pons and midbrain), right orbitofrontal cortex, and bilateral cuneus cortices merit further investigations.

## Methods

### Identification, selection, and quality assessment of studies

We comprehensively searched PubMed, Embase, and Web of Science databases for studies published between January 1st, 2000 and June 24, 2016 using the following keywords “Parkinson” OR “Parkinson’s disease”, AND “amplitude of low frequency fluctuations” OR “ALFF”. Other resources, such as the reference lists of included studies and relevant review articles were additionally identified. Studies were included if they satisfied the following conditions: (i) they were published in English as an original article in a peer-reviewed journal; (ii) they reported ALFF results at the whole-brain level for direct comparison between patients with idiopathic PD and healthy controls; (iii) they reported negative results or three-dimensional coordinates in a standardized stereotaxic space for regions with significant differences; (iv) they reported significant results using thresholds for significance corrected for multiple comparisons or uncorrected with spatial extent thresholds. In order to reduce the heterogeneity, studies were excluded if they explicitly indicated patients with PD diagnosed with comorbid neurological or psychiatric disorders (i.e., depression, visual hallucinations, or cognitive impairment) or if they only reported the on-state results. The baseline result was included if the study was longitudinal. In cases where multiple articles were identified to use the overlapped patient datasets, the one with the largest sample size and the most comprehensive information was selected. The quality of each study included in this meta-analysis was assessed according to a 20-point checklist developed for rs-fMRI studies^30^ (see Supplementary Table 2). Literature search, study assessment and selection, and data extraction with a standard form were independently performed by two authors (P.L.P and Y.Z.). Any discrepancies were discussed with a third researcher for a final decision (Y.L.). The Meta-analysis Of Observational Studies in Epidemiology (MOOSE) guidelines were followed in this study^86^.

## Data analysis

### Voxel-wise meta-analysis

This voxel-wise meta-analysis was carried out using the SDM software package available at http://www.sdmproject.com. The details of the approach have been described elsewhere^28^,^31^,^32^,^33^ First, we extracted peak coordinates and effect sizes (*t*-values) of ALFF differences between patients with PD and healthy controls from each dataset. Second, an effect-size signed map of the ALFF differences was then separately recreated for each dataset. SDM calculates both positive and negative differences between datasets in the same map^31^,^33^. And third, the mean map was generated by voxel-wise calculation of the random-effects mean of the dataset maps, which was weighted by the sample size, intra-dataset variability, and additional between-dataset heterogeneity. Default SDM settings (full width at half maximum [FWHM] = 20 mm, p = 0.005, peak height Z = 1, cluster extent = 10 voxels), which were found to optimally balance false positives and negatives, were applied to the results^31^,^33^.

### Analyses of jackknife sensitivity, heterogeneity, and publication bias

To assess the replicability of the results, a jackknife sensitivity analysis was performed by iteratively repeating the same analysis, excluding one dataset each time^28^,^31^.

In addition, a heterogeneity analysis was conducted using a random effects model with Q statistics to explore unexplained between-study variability in the results. These analyses were thresholded with the default SDM settings (FWHM = 20 mm, p = 0.005, peak height Z = 1, cluster extent = 10 voxels)^31^,^33^.

In order to evaluate possible publication bias, the Stata/SE 12.0 software for Windows (Stata Corp LP, College Station, TX, USA) was used to perform the Egger’s test by extracting the values from the peak coordinates of the voxel-wise meta-analysis between patients with PD and healthy controls^87^. Statistical significance was thresholded at a p-value less than 0.05.

The Stata/SE 12.0 software was additionally used to meta-analyze mean age and gender distribution in the PD groups and control groups across studies with a random-effects model to test whether there is heterogeneity.

### Meta-regression analyses

Meta-regression analyses were carried out to explore the effects of age, illness severity (Hoehn and Yahr [H&Y] stage), motor severity (Unified Parkinson’s Disease Rating Scale (UPDRS) Part III [UPDRS-III] score), and illness duration, which could potentially influence the analytic results. P-value less than 0.0005 and cluster extent more than 10 voxels were considered statistically significant^31^,^32^.

## Additional Information

**How to cite this article:** Pan, P. L. *et al*. Abnormalities of regional brain function in Parkinson's disease: a meta-analysis of resting state functional magnetic resonance imaging studies. *Sci. Rep.* **7**, 40469; doi: 10.1038/srep40469 (2017).

**Publisher's note:** Springer Nature remains neutral with regard to jurisdictional claims in published maps and institutional affiliations.

## Supplementary Material

Supplementary Information

## Figures and Tables

**Table 1:** Characteristics of ALFF studies included in the meta-analysis.

Study	Sample (female)	Mean Age (SD)	UPDRS-III (SD)	H&Y stage (SD)	Duration (SD)	Medication status	Scanner	Software	FWHM	Threshold	Quality scores^#^
Kwak *et al*.^25^	PD 24 (2) HC 24 (5)	64.3 (8) 63.3 (7)	18.5 (8)	2.2 (0.3)	5.4 (3)	Off-state	3.0 T	SPM5	8 mm	p < 0.001 uncorrected	20
Wen *et al*.^19^	PD 16 (8) HC 21 (8)	60.7 (18.7) 55.4 (16.4)	33.8 (24.2)	1.5 (1)	5.6 (7.4)	Off-state	3.0 T	SPM8, REST	5 mm	p < 0.005 uncorrected	20
Possin *et al*.^23^	PD 12 (9) HC 15 (11)	73.9 (5.9) 72.9 (5.2)	30.8 (14.5)	NA	9 (7)	Off-state	3.0 T	SPM8, REST	4 mm	p < 0.05 corrected	19
Skidmore *et al*.^13^	PD 14 (3) HC 15 (6)	62 (9) 65 (13)	37 (13)	NA	NA	Off-state	3.0 T	AFNI	6 mm	p < 0.005 uncorrected	18
Zhang *et al*.^24^	PD 72 (37) HC 78 (32)	59.7 (11.9) 58.6 (8.5)	20.24 (8.44)	NA	7.05 (6.01)	Off-state	3.0 T	SPM8, REST	8 mm	p < 0.05 corrected	19
Hou, *et al*.^26^	PD 101 (42) HC 102 (42)	59.84 (7.15) 59.91 (7.09)	25.54 (11.51)	1.87 (0.71)	7.23 (4.42)	Off-state	3.0 T	SPM8, DPARSF	3 mm	p < 0.05 corrected	20
Luo *et al*.^21^	PD 30 (15) HC 30 (15)	53.64 (10.18) 51.9 (7.70)	26.83 (12.44)	1.73 (0.38)	2.12 (1.30)	Off-state	3.0 T	SPM8, REST	8 mm	p < 0.001 corrected	20
Hu *et al*.^17^	PD 17 (7) HC 20 (9)	60.29 (12.03) 58.48 (6.89)	17.11 (6.12)	NA	3.94 (2.57)	Off-state	3.0 T	SPM8, REST	8 mm	p < 0.005 uncorrected	19
Chen *et al*.^15^	PD^1^ 12 (8) PD^2^ 19 (7) HC 22 (10)	62.6 (8.71) 64.8 (8.34) 65.1 (5.00)	19.1 (11.5) 21.6 (11.6)	1.87 (0.607) 2.13 (0.984)	6.38 (4.01) 6.68 (4.85)	Off-state	3.0 T	SPM8, REST	4 mm	p < 0.05 corrected	20
Luo *et al*.^18^	PD^3^ 28 (14) PD^4^ 28 (14) PD^5^ 24 (11) HC 30 (15)	52.45 (9.18) 54.12 (8.24) 54.41 (10.59) 53.53 (10.45)	14.11 (6.45) 29.68 (8.22) 43.54 (12.65)	1.0 (NA) 2.0 (NA) 3.0 (NA)	1.56 (1.42) 3.78 (2.87) 5.31 (4.77)	Off-state	3.0 T	SPM5, REST	8 mm	p < 0.001 uncorrected	19
Xiang *et al*.^27^	PD 24 (12) HC 24 (12)	62.7 (7.4) 65.6 (6.9)	22.0 (7.0)	2.2 (0.9)	7.0 (3.3)	Off-state	3.0 T	SPM8, REST	8 mm	p < 0.01 corrected	20

**Key:** ALFF, amplitude of low-frequency fluctuations; PD, Parkinson’s disease; HC, healthy controls; SD, standard deviation; UPDRS-III, Unified Parkinson’s Disease Rating Scale, Part III; H&Y, Hoehn and Yahr disability scale; FWHM, full width at half maximum; NA, not available; AFNI, Analysis of Functional NeuroImage software; fMRI, functional magnetic resonance imaging; SPM, statistical parametric mapping; REST, the Resting-State fMRI Data Analysis Toolkit; ^1^postural instability/gait difficulty subtype of PD; ^2^tremor-dominant subtype of PD; ^3^PD patients at H&Y stage I; ^4^PD patients at H&Y stage II; ^5^PD patients at H&Y stage III; ^#^a maximum score of 20 for each study.

**Table 2:** ALFF differences in patients with PD compared to healthy controls.

Cluster	Anatomical label	Peak MNI coordinate (x, y, z)	No. of voxels	SDM-Z value	p value	Heterogeneity	Egger’s test
PD > HC							
A	Right inferior temporal/middle temporal/fusiform parahippocampal gyri (BA 20)	44, −18, −30	790	3.00	0.000015	Yes	＜0.001
B	Right inferior parietal gyrus (BAs 39, 40, and 7)	36, −46, 52	740	2.81	0.000062	No	0.172
C	Brainstem (pons and midbrain)	2, −32, −26	51	2.16	0.0011	Yes	0.046
D	Right orbitofrontal cortex (BA 11)	14, 18, −16	46	1.92	0.0029	No	0.018
PD < HC							
E	Left/Right cuneus cortices (BAs 18, 17, and 19)	0, −90, 14	641	−3.03	0.000040	No	0.002
F	Left/Right supplementary motor areas (BA 6)	0, 0, 62	352	−2.44	0.00080	No	0.500
G	Left putamen	−26, 8, 2	187	−2.45	0.00078	No	0.186
H	Left cuneus cortex (BA 23)	−18, −58, 26	43	−2.21	0.0018	Yes	0.140
I	Left inferior parietal gyrus (BAs 40 and 2)	−48, −34, 38	30	−2.07	0.0030	No	0.065
J	Left lateral premotor cortex (BA 6)	−44, 0, 44	26	−2.07	0.0029	No	0.058

**Key:** ALFF, amplitude of low-frequency fluctuations; PD, Parkinson’s disease; HC, healthy controls; MNI, Montreal Neurological Institute; BA, Brodmann area; No., number; SDM, Seed-based *d* Mapping.

**Table 3:** Jackknife sensitivity analysis.

All studies but …	A	B	C	D	E	F	G	H	I	J
Kwak *et al*.^25^	Yes	Yes	Yes	Yes	Yes	Yes	Yes	Yes	Yes	Yes
Wen *et al*.^19^	Yes	Yes	Yes	Yes	Yes	Yes	Yes	Yes	Yes	Yes
Possin *et al*.^23^	Yes	Yes	Yes	No	Yes	Yes	Yes	No	Yes	No
Skidmore *et al*.^13^	Yes	Yes	Yes	Yes	Yes	Yes	Yes	Yes	Yes	Yes
Zhang *et al*.^24^	Yes	Yes	Yes	Yes	Yes	Yes	Yes	Yes	Yes	Yes
Hou, *et al*.^26^	Yes	Yes	No	No	Yes	No	No	Yes	No	No
Luo *et al*.^21^	Yes	Yes	Yes	Yes	Yes	Yes	Yes	Yes	Yes	Yes
Hu *et al*.^17^	Yes	Yes	Yes	Yes	Yes	Yes	Yes	Yes	Yes	Yes
Chen *et al*.^a ^15	Yes	Yes	No	Yes	Yes	Yes	Yes	Yes	Yes	Yes
Chen *et al*.^b ^15	Yes	Yes	Yes	Yes	Yes	Yes	Yes	Yes	Yes	Yes
Luo *et al*.^c ^18	Yes	Yes	Yes	Yes	Yes	Yes	Yes	No	Yes	Yes
Luo *et al*.^d ^18	Yes	Yes	Yes	Yes	Yes	Yes	Yes	No	Yes	Yes
Luo *et al*.^e ^18	Yes	Yes	Yes	Yes	Yes	Yes	Yes	No	Yes	Yes
Xiang *et al*.^27^	Yes	Yes	Yes	Yes	Yes	Yes	Yes	Yes	Yes	Yes
Total	14 out of 14	14 out of 14	12 out of 14	12 out of 14	14 out of 14	13 out of 14	13 out of 14	10 out of 14	13 out of 14	12 out of 14

**Key:** A, Right inferior temporal/middle temporal/fusiform/parahippocampal gyri; B, Right inferior parietal gyrus; C, Brainstem (pons and midbrain); D, Right orbitofrontal cortex; E, Left/Right cuneus cortices; F, Left/Right supplementary motor areas; G, Left putamen; H, Left cuneus cortex; I, Left inferior parietal gyrus; J, Left lateral premotor cortex; Yes, the region reported; No, the region not reported; ^a^postural instability/gait difficulty subtype of PD; ^b^tremor-dominant subtype of PD; ^c^PD patients at H&Y stage I; ^d^PD patients at H&Y stage II; ^e^PD patients at H&Y stage III.

**Table 4:** Meta-regression analyses.

Anatomical label	Peak MNI coordinate (x, y, z)	No. of voxels	SDM-Z value	p value
**Effects of age: ALFF changes in studies with older patients compared to younger patients**				
Right inferior temporal/middle temporal/fusiform/parahippocampal gyri (BA 20)	44, −16, −28	169	2.193	0.0000305
Right/Left cuneus cortices (BAs 18 and 17)	6, −98, 10	106	−2.695	0.0000361
**Effects of motor severity: ALFF changes in studies with higher average UPDRS-III score**				
Right inferior parietal gyrus (BAs 39, 40, and 7)	40, −50, 48	811	3.567	~0
Left/Right precuneus/PCC (BAs 23 and 30)	−16, −90, 38	61	2.759	0.0000439
Right/Left supplementary motor area (BA 6)	2, 10, 60	223	−2.114	0.000150
**Effects of illness severity: ALFF changes in studies with higher average H&Y stage**				
Left/Right precuneus/PCC (BAs 23 and 30)	−4, −46, 32	322	2.405	0.000214
Right inferior parietal gyrus (BAs 39, 40, and 7)	38, −54, 46	200	2.643	0.0000718
**Effects of illness duration: ALFF changes in studies with longer average illness duration**				
Right fusiform/inferior temporal gyri (BA 20)	40, −20, −28	125	3.830	0.0000279

**Key:** ALFF, amplitude of low-frequency fluctuations; PD, Parkinson’s disease; MNI, Montreal Neurological Institute; No., number; SDM, Seed-based *d* Mapping; BA, Brodmann area; UPDRS-III, Unified Parkinson’s Disease Rating Scale, Part III; H&Y, Hoehn and Yahr disability scale; PCC, posterior cingulate cortex.
