# Supplementary material for: Abnormalities of regional brain function in Parkinson’s disease: a meta-analysis of resting state functional magnetic resonance imaging studies
Source: Sci Rep. 2017 Jan 12;7:40469. doi: 10.1038/srep40469 (PMC5228032; doi:10.1038/srep40469)
Supplement: Supplementary Information [file srep40469-s1.pdf]

Supplementary Information

Title: Abnormalities of regional brain function in Parkinson's disease: a meta-analysis of resting state functional magnetic resonance imaging studies

Authors: PingLei Pan, Yang Zhang, Yi Liu, He Zhang, DeNing Guan, and Yun Xu

Supplementary Table 1. Main findings from the original studies

Study	Main findings at the off-state (PD patients relative to healthy controls)
Kwak et al. (2012)[1]	Decreased ALFFs in PD in the primary and secondary motor areas, and inferior temporal gyrus; Increased ALFFs in PD the precuneus, cerebellum lobule V, and superior and middle temporal gyri
Wen et al. (2013)[2]	Decreased ALFFs in non-depressed PD in the bilateral caudate, left putamen, supplementary motor area (SMA) , bilateral superior frontal gyrus and posterior cingulate cortex; Increased ALFFs in non-depressed PD in the left middle temporal gyrus, right middle occipital gyrus, bilateral superior occipital gyrus, left inferior temporal gyrus, left precuneus, and right angular gyrus
Possin et al. (2013)[3]	Decreased fALFFs in PD in the frontal, temporal, parietal and occipital lobes, subcortical regions, and cerebellum
Skidmore et al. (2013)[4]	Decreased ALFFs in PD in the right mesial frontal gyrus, left SMA, left inferior occipital gyrus, left middle frontal gyrus, and right inferior cerebellum (lobules VII and VIII); Increased ALFFs in PD in the left superior cerebellum (lobules IV and V)
Zhang et al. (2013)[5]	Decreased ALFFs (slow-5) in PD in the cerebellum, left middle temporal gyrus, left middle occipital gyrus, right cuneus, and left calcarine; Increased ALFFs in PD in the bilateral caudate, right hippocampus, left superior and inferior temporal gyrus, right fusiform, and right inferior frontal gyrus
	Decreased ALFFs (slow-4) in PD in the cerebellum, left thalamus, left middle occipital gyrus, and right inferior occipital gyrus
Hou, et al. (2014)[6]	Decreased ALFFs (slow-5) in PD in the left precentral gyrus, left pre-SMA, left superior temporal gyrus, left medial frontal gyrus and left putamen; Increased ALFFs in PD in the right inferior temporal gyrus, left cerebellum, right cerebellum, and right midbrain
	Decreased ALFFs (slow-4) in PD in the left precentral gyrus, left pre-SMA, right occipital Lobe, left superior temporal gyrus, bilateral putamen; Increased ALFFs in PD in the right inferior parietal lobule, right fusiform gyrus, right inferior frontal gyrus, left cerebellum, right cerebellum, right midbrain, and right pons

Luo et al. (2014)[7]	No significant ALFF difference between the non-depressed PD patients and normal controls
Hu et al. (2015)[8]	Increased fALFF in PD in the left inferior temporal gyrus, right inferior parietal lobule and right middle frontal gyrus
Chen al. (2015)[9]	Increased ALFFs in tremor-dominant subtype of PD the right cerebellar lobule VIII and the right superior frontal gyrus
	Decreased ALFFs in postural instability/gait difficulty subtype of PD in the bilateral putamen, left cerebellum (crus II), right cerebellum(lobule VIII), and cerebellar vermis VIII; Increased ALFFs in postural instability/gait difficulty subtype of PD in left cerebellum (the lobule III, IV, and V) and some cortical areas, including the right inferior and superior temporal gyrus, the left superior frontal, and parietal gyrus
Luo et al. (2015)[10]	Decreased ALFFs in PD patients at stage I in the left occipital cortex and left lingual gyrus
	Decreased ALFFs in PD patients at stage II in the left occipital cortex and left lingual gyrus; Increased ALFFs in PD patients at stage II in the posterior cingulate cortex and right lateral parietal cortex
	Decreased ALFFs in PD patients at stage III in the left occipital cortex and left lingual gyrus; Increased ALFFs in PD patients at stage III in the posterior cingulate cortex and right lateral parietal cortex
Xiang et al. (2016)[11]	Decreased ALFFs in PD patients in the left thalamus, caudate, and right insula/inferior prefrontal gyrus; Increased ALFFs in PD patients in the right medial prefrontal cortex and right dorsolateral prefrontal cortex

Key: PD, Parkinson's disease; ALFF, amplitude of low frequency fluctuations; fALFF, fractional ALFF

References

1 Kwak, Y. *et al.* L-DOPA changes spontaneous low-frequency BOLD signal oscillations in Parkinson's disease: a resting state fMRI study. *Frontiers in systems neuroscience* **6**, 52, doi:10.3389/fnsys.2012.00052 (2012).

2 Wen, X., Wu, X., Liu, J., Li, K. & Yao, L. Abnormal baseline brain activity in non-depressed Parkinson's disease and depressed Parkinson's disease: a resting-state functional magnetic resonance imaging study. *PloS one* **8**, e63691, doi:10.1371/journal.pone.0063691 (2013).

3 Possin, K. L. *et al.* Rivastigmine is associated with restoration of left frontal brain activity in Parkinson's disease. *Movement Disorders* **28**, 1384-1390 (2013).

4 Skidmore, F. M. *et al.* Reliability analysis of the resting state can sensitively and specifically identify the presence of Parkinson disease. *NeuroImage* **75**, 249-261, doi:10.1016/j.neuroimage.2011.06.056 (2013).

5 Zhang, J. *et al.* Specific frequency band of amplitude low-frequency fluctuation predicts Parkinson's disease. *Behavioural brain research* **252**, 18-23, doi:10.1016/j.bbr.2013.05.039 (2013).

6 Hou, Y., Wu, X., Hallett, M., Chan, P. & Wu, T. Frequency-dependent neural activity in Parkinson's disease. *Human brain mapping* **35**, 5815-5833, doi:10.1002/hbm.22587 (2014).

7 Luo, C. *et al.* Resting-state fMRI study on drug-naive patients with Parkinson's disease and with depression. *Journal of neurology, neurosurgery, and psychiatry* **85**, 675-683, doi:10.1136/jnnp-2013-306237 (2014).

8 Hu, X. *et al.* Altered Resting-State Brain Activity and Connectivity in Depressed Parkinson's Disease. *PloS one* **10**, e0131133, doi:10.1371/journal.pone.0131133 (2015).

9 Chen, H. M. *et al.* Different patterns of spontaneous brain activity between tremor-dominant and postural instability/gait difficulty subtypes of Parkinson's disease: a resting-state fMRI study. *CNS neuroscience & therapeutics* **21**, 855-866, doi:10.1111/cns.12464 (2015).

10 Luo, C. *et al.* The trajectory of disturbed resting-state cerebral function in Parkinson's disease at different Hoehn and Yahr stages. *Human brain mapping* **36**, 3104-3116, doi:10.1002/hbm.22831 (2015).

11 Xiang, J. *et al.* Altered Spontaneous Brain Activity in Cortical and Subcortical Regions in Parkinson's Disease. *Parkinson's disease* **2016**, 5246021, doi:10.1155/2016/5246021 (2016).

Supplementary Table 2. Criteria for objective assessment of methodological quality of individual studies

**Category 1: Sample characteristics (10)**
1. Patients were evaluated with specific standardized diagnostic criteria (1)
2. Important demographic data (age and gender) were reported with mean (or median) and standard deviations (or range)) (2)
3. Healthy comparison subjects were evaluated to exclude psychiatric and medical illnesses and demographic data was reported (1)
4. Important clinical variables (e.g. illness duration, medication status, UPDRS III score, H&Y stage) were reported with mean (or median) and standard deviations (or range)) (4)
5. Sample size per group > 10 (2)
**Category 2: Methodology and reporting (10)**
1. Whole brain analysis was automated without a priori regional selection (3)
2. Magnet strength at least 1.5T (1)
3. At least 5 minutes of resting state acquisition (1)
4. Whole brain coverage of resting scans (1)
5. The acquisition and preprocessing techniques were clearly described so that they could be reproduced (1)
6. Coordinates reported in a standard space (1)
7. Significant results are reported after correction for multiple testing using a standard statistical procedure (FDR, FWE or permutation-based methods) (1)
8. Conclusions were consistent with the results obtained and the limitations were discussed (1)

Key: A maximum score of 20 for each study, allocated as per the criteria specified above.

Supplementary Table 3. Regions of ALFF heterogeneity from the SDM analysis

Anatomical regions	Maximum MNI coordinate	Voxels	SDM-Z	p
Left superior/middle temporal gyri/insula (BAs 48 and 22)	-46, -12, -6	547	4.402	0.0000268
Right caudate nucleus (BA 25)	12, 20, 4	252	4.615	0.0000150
Right fusiform /inferior temporal gyri (BA 20)	44, -26, -24	106	3.354	0.000385
Right cerebellum (lobule VI)	4, -70, -16	108	2.991	0.000810
Right brain stem	2, -16, -12	61	3.578	0.000237
Left cerebellum (crus I)	-30, -78, -22	63	2.825	0.00113
Right striatum	22, 16, 0	49	3.740	0.000159
Right inferior frontal gyrus (BA 45)	52, 26, 8	42	2.388	0.00254
Left superior occipital gyrus (BAs18 and 19)	-18, -92, 28	38	2.893	0.000987
Left cuneus cortex (BA 23)	-18, -58, 24	30	2.862	0.000811
Right inferior temporal gyrus (BA 20)	60, -24, -26	28	2.727	0.00136
Right cerebellum (lobule IV/V)	10, -58, -16	18	2.476	0.00216
Left posterior cingulate cortex (BA 23)	-4, -44, 34	18	2.087	0.00345

Key: ALFF, amplitude of low-frequency fluctuations; MNI, Montreal Neurological Institute; SDM, Seed-based *d* Mapping; BA, Brodmann area
